# Macrophage Migration Inhibitory Factor (MIF) Inhibition in a Murine Model of Bleomycin-Induced Pulmonary Fibrosis

**DOI:** 10.3390/ijms19124105

**Published:** 2018-12-18

**Authors:** Sven Günther, Jennifer Bordenave, Thông Hua-Huy, Carole Nicco, Amélie Cumont, Raphaël Thuillet, Ly Tu, Timothée Quatremarre, Thomas Guilbert, Gaël Jalce, Frédéric Batteux, Marc Humbert, Laurent Savale, Christophe Guignabert, Anh-Tuan Dinh-Xuan

**Affiliations:** 1 National Institute for Health and Medical Research (INSERM) UMR_S 1016, Cochin Institute, 75014 Paris, France; sven.gunther@aphp.fr (S.G.); huythonghua@yahoo.com (T.H.-H.); carole.nicco@parisdescartes.fr (C.N.); thomas.guilbert@inserm.fr (T.G.); frederic.batteux@aphp.fr (F.B.); anh-tuan.dinh-xuan@aphp.fr (A.-T.D.X.); 2 Université Paris-Descartes, Sorbonne Paris Cité, 75014 Paris, France; 3 Service de Physiologie-Explorations Fonctionnelles, Hôpital Cochin, Assistance Publique-Hôpitaux de Paris (AP-HP), 75014 Paris, France; 4 INSERM UMR_S 999, Hôpital Marie Lannelongue, 92350 Le Plessis-Robinson, France; jennifer.bordenave@inserm.fr (J.B.); amelie.cumont@laposte.net (A.C.); raphael.thuillet@inserm.fr (R.T.); ly.tu@inserm.fr (L.T.); timothee.quatremare@inserm.fr (T.Q.); marc.humbert@bct.aphp.fr (M.H.); laurent.savale@gmail.com (L.S.); 5 Faculté de Médecine, Université Paris-Sud, Université Paris-Saclay, 94270 Le Kremlin-Bicêtre, France; 6 National Centre for Scientific Research (CNRS) UMR 8104, 75014 Paris, France; 7 Apaxen, 6041 Gosselies, Belgique; gael.jalce@apaxen.com; 8 Service de Pneumologie, Centre de Référence de l’Hypertension Pulmonaire, DHU Thorax Innovation, Hôpital Bicêtre, Assistance Publique-Hôpitaux de Paris (AP-HP), 94270 Le Kremlin-Bicêtre, France

**Keywords:** idiopathic pulmonary fibrosis associated with pulmonary hypertension (IPF-PH), pulmonary vascular remodeling, molecular target, macrophage migration inhibitory factor

## Abstract

Background: Pulmonary hypertension (PH) is a common complication of idiopathic pulmonary fibrosis (IPF) that significantly contributes to morbidity and mortality. Macrophage migration inhibitory factor (MIF) is a critical factor in vascular remodeling of the pulmonary circulation. Objectives: We tested the effects of two small molecules targeting MIF on bleomycin (BLM)-induced collagen deposition, PH, and vascular remodeling in mouse lungs. Methods: We examined the distribution pattern of MIF, CD74, and CXCR4 in the lungs of patients with IPF-PH and the lungs of BLM-injected mice. Then, treatments were realized with (*S*,*R*)-3-(4-hydroxyphenyl)-4,5-dihydro-5-isoxazole acetic acid methyl ester (ISO-1) and *N*-(3-hydroxy-4-fluorobenzyl)-5 trifluoromethylbenzoxazol-2-thione **31** (20 mg/kg/day per os for 3 weeks) started 24 h after an intratracheal BLM administration. Results: More intense immunoreactivity was noted for MIF, CD74, and CXCR4 in lungs from IPF-PH patients and BLM-injected mice. Furthermore, we found that treatments of BLM-injected mice with ISO-1 or compound **31** attenuated lung collagen deposition and right ventricular systolic pressure increase. Additionally, reduced pulmonary inflammatory infiltration and pulmonary arterial muscularization were observed in the lungs of BLM-injected mice treated with ISO-1 or compound **31**. Conclusions: Treatments with ISO-1 or compound **31** attenuates BLM-induced inflammation and fibrosis in lung, and prevents PH development in mice, suggesting that MIF is an important factor for IPF-PH development.

## 1. Introduction

Idiopathic pulmonary fibrosis (IPF) is a progressive diffuse parenchymal disease with poor prognosis characterized by the progressive scarring of lung tissue [1]. Pulmonary hypertension (PH) often complicates the course of IPF, and has a strong association with functional impairment and worse outcomes, especially when PH is detected late [2]. Mechanisms contributing to pulmonary vascular remodeling in IPF are complex, and diverse molecules are involved, including different cytokines and growth factors [3].

Emerging evidence suggests that the macrophage migration inhibitory factor (MIF), one of the oldest known immunological mediators, could play a role in the pathogenesis of IPF. MIF was identified by a proteomic approach in the bronchoalveolar lavage (BAL) of patients with IPF [4], and the strong immunoreactivity of MIF was reported to co-localize in actively fibrosing areas, such as fibroblast foci and lung remodeling zones [5]. Furthermore, MIF levels in lung tissues and BAL fluids were significantly increased in the murine model of bleomycin (BLM)-induced pulmonary fibrosis and chronic treatment with an anti-MIF antibody was reported to have a beneficial effect on lung inflammation in the acute phase, which is the period of 5–10 days after BLM administration [6]. Interestingly, exposure of lung fibroblasts to bleomycin, a known inducer of fibrosis, resulted in an increase in MIF secretion to a level close to that found in fibroblasts derived from the lungs of patients with limited (lSSc) and diffuse (dSSc) systemic sclerosis [7]. Finally, circulating MIF levels have been recently identified as a critical factor in the vascular remodeling and homeostasis of pulmonary circulation [8,9].

Therefore, we examined the expression and distribution pattern of MIF and its two receptors, CD74 and CXCR4, in the lungs of patients with IPF-PH, and tested the effects of ISO-1 and compound **31**, a new small molecule targeting MIF, on BLM-induced collagen deposition, PH, and vascular remodeling in mouse lungs.

## 2. Results

### 2.1. MIF and Its Two Main Receptors, CD74 and CXCR4, Are Upregulated in Lungs from Patients with IPF Associated with PH (IPF-PH)

**Figure 1:**
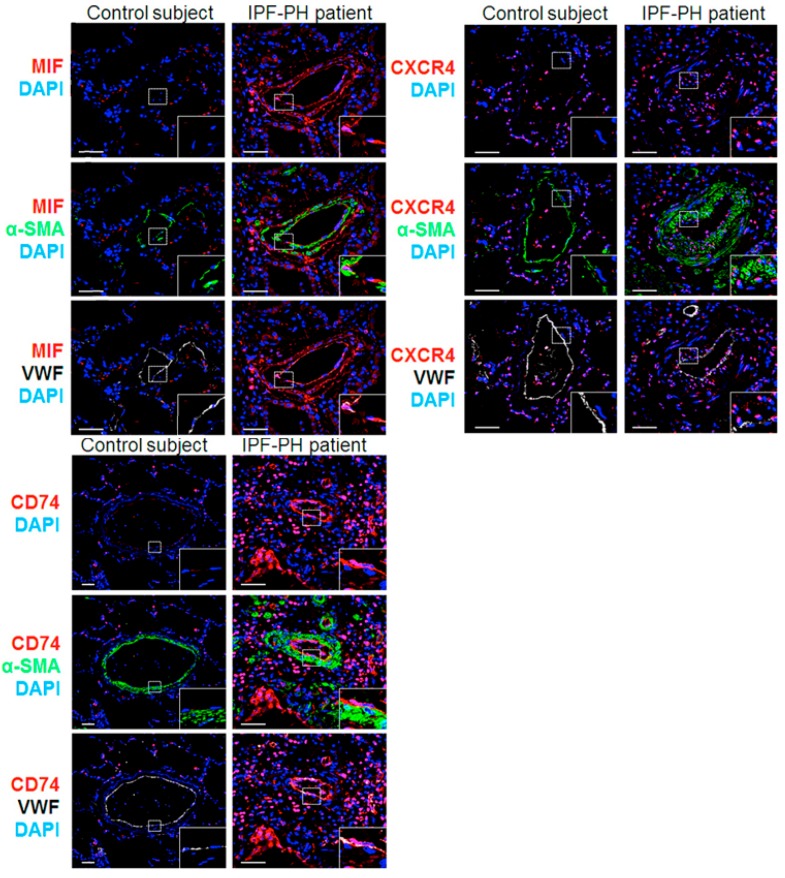
Increased expressions of MIF, CXCR4, and CD74 in lungs of patients with idiopathic pulmonary fibrosis with pulmonary hypertension (IPF-PH) and control subjects. Representative images of MIF (red; upper panel), CXCR4 (red; middle panel), and CD74 (red; lower panel) staining with α-smooth muscle actin (α-SMA; green) or von Willebrand factor (vWF; white) and DAPI in lungs from control subjects (*n* = 3) and IPF-PH (*n* = 3). Scale bar = 20 μm in all sections. DAPI = 4′,6-diamidino-2-phenylindole.

In a first step, we analyzed and compared the expression patterns of MIF and its CD74 and CXCR4 receptors in lung specimens of control and IPF-PH patients (Figure 1). We found higher numbers of cells positive for MIF or its two main receptors, CD74 and CXCR4, in paraffin-embedded lungs of IPF-PH patients, as compared with control lungs (Figure 1). In lungs from IPF-PH patients, MIF CXCR4 and CD74 positive cells were observed in the perivascular area and within the vascular wall (Figure 1). In addition, our immunofluorescent studies indicated that fibroblast foci from IPF-PH patients expressed low levels of MIF and CXCR4, but no CD74 (Supplementary Figure S1).

### 2.2. MIF, CD74, and CXCR4 Protein Levels Are Increased in Lungs from Bleomycin-Injected Mice

**Figure 2:**
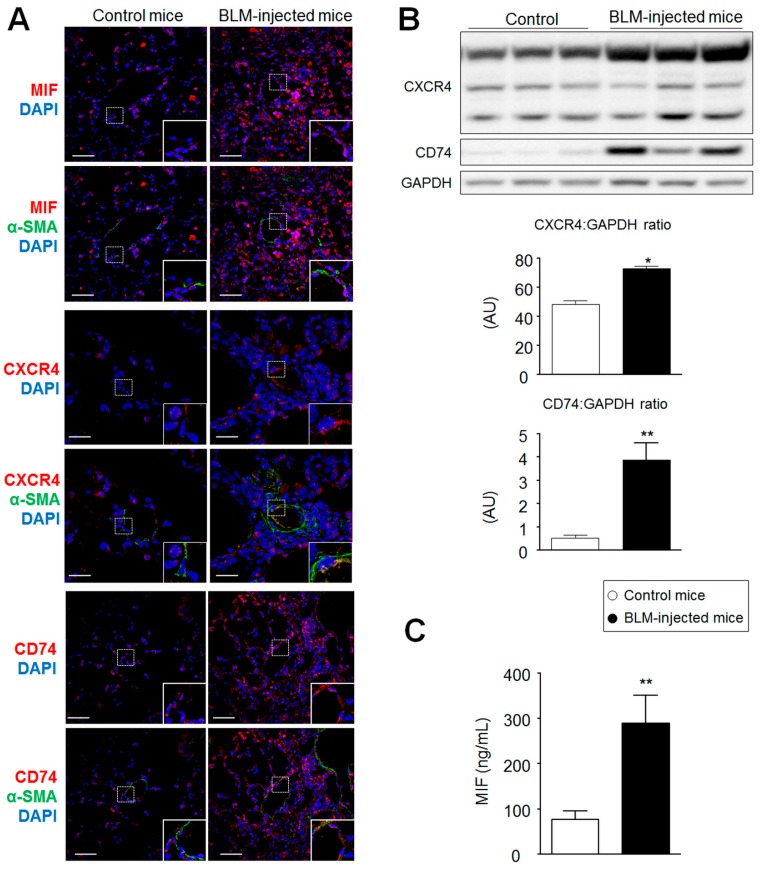
Increased expression of macrophage migration inhibitory factor (MIF), CXCR4, and CD74 in lungs from bleomycin (BLM)-injected mice. (**A**) Representative images of MIF (red; upper panel), CXCR4 (red; middle panel), and CD74 (red; lower panel) staining with α-smooth muscle actin (α-SMA; green) and DAPI (blue) in lungs from control and BLM-injected mice. (**B**) Representative Western blots and quantification of the CXCR4/GAPDH and CD74/GAPDH ratios in lungs from control and BLM-injected mice. (**C**) Levels of circulating MIF proteins in the serum of BLM-injected mice compared to those of control mice. Values are means ± SEM (*n* = 4–8), where *n* represents the number of mice. * p < 0.05; ** p < 0.01 compared to control mice. Scale bar = 20 μm in all sections. AU = arbitrary unit; DAPI = 4′,6-diamidino-2-phenylindole.

Although the animal model of BLM-induced lung fibrosis does not completely reproduce the complex human disease [10,11], this model is widely used to evaluate the antifibrotic efficacy of molecules against lung fibrosis [12,13,14]. Therefore, we conducted parallel immunohistochemical evaluations to investigate whether the expression of MIF and its two main receptors were modified in the lungs of BLM-injected mice (Figure 2). Consistent with our observations in human lung specimens, we found marked increases in MIF, CXCR4, and CD74 protein levels in lungs isolated 21 days post-challenge (Figure 2A,B) associated with a 2–3-fold increase in MIF serum protein levels (Figure 2C).

### 2.3. Chronic Treatment with ISO-1 and Compound ***31*** Attenuates Extracellular Matrix Deposition in Lungs of Bleomycin-Injected Mice

**Figure 3:**
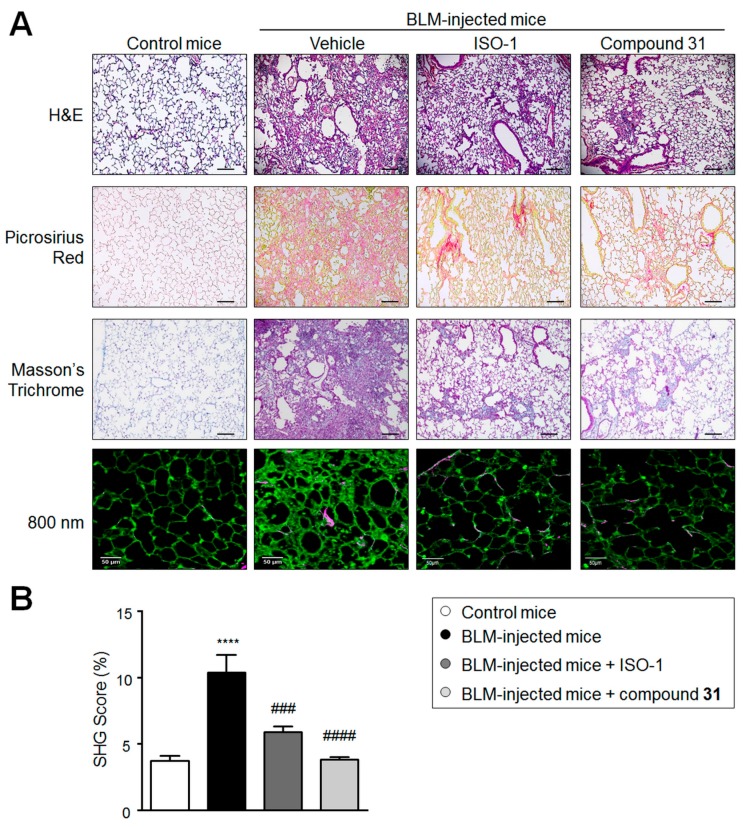
Chronic treatment with ISO-1 and compound **31** attenuates matrix deposition in lungs from bleomycin (BLM)-injected mice. (**A**) Representative images of hematoxylin eosin (H&E), Picrosirius red, or Masson’s trichrome staining and analysis of second harmonic generation (SHG) of radiations with a fundamental wavelength of 800 nm, and (**B**) quantification of the SHG score in lungs from control and BLM-injected mice. Values are means ± SEM (*n* = 4–8), where *n* represents the number of mice. **** p < 0.001 compared to control mice; *### p <* 0.001 and *#### p <* 0.0001 compared to BLM-injected mice. Scale bar = 50 μm in all sections.

To determine the effects of treatments on extracellular matrix composition changes three weeks after lung fibrosis induction, Masson’s trichrome, Picrosirius red staining, and second harmonic generation (SHG) microscopy were used to examine the collagen density and structure in the lungs of BLM-treated mice (Figure 3). On day 21, in BLM-injected mice treated with vehicle, a markedly increased collagen deposition and SHG score were observed in lungs when compared to controls (Figure 3A,B). By contrast, both Picrosirius red and Masson’s trichrome staining and the SHG scores indicated a substantial reduction in collagen deposition in the lungs of BLM-injected mice treated with ISO-1 or compound **31** when compared to BLM-injected mice treated with vehicle (Figure 3A,B).

### 2.4. Daily Treatment with ISO-1 and Compound ***31*** Prevents the Development of Pulmonary Hypertension (PH) in Lungs of Bleomycin-Injected Mice

**Figure 4:**
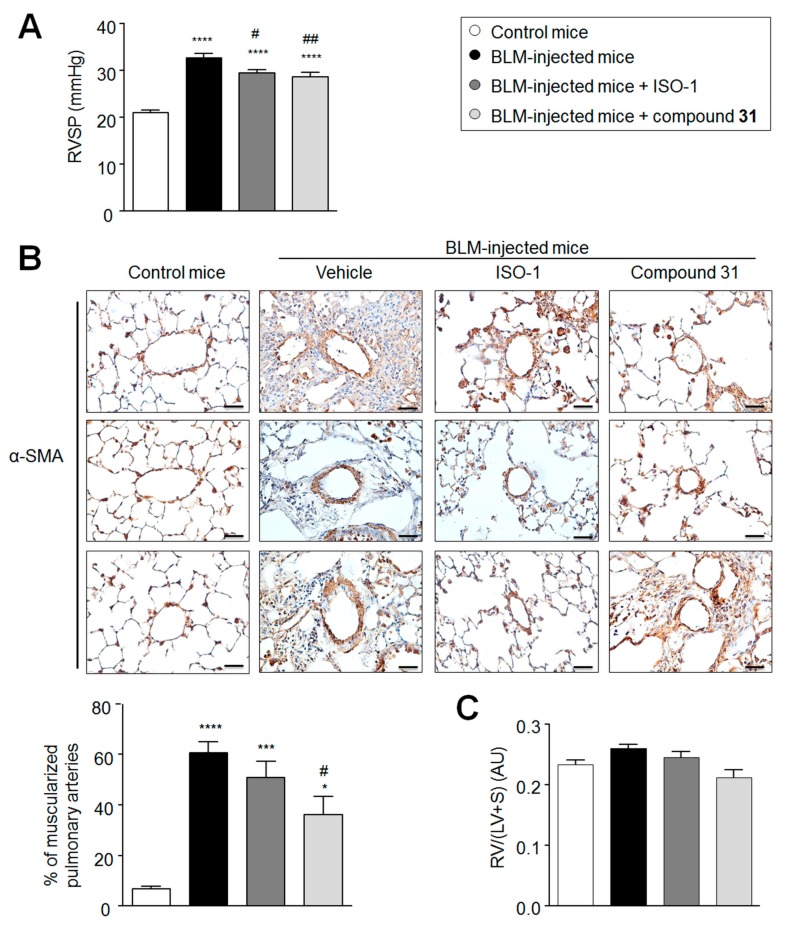
Chronic treatment with ISO-1 and compound **31** prevents the development of pulmonary hypertension in bleomycin (BLM)-injected mice. (**A**) Right ventricular systolic pressure (RVSP) in control or BLM-injected mice treated, or not, with ISO-1 or compound **31**. (**B**) Representative images of α-smooth muscle actin (α-SMA) staining and quantification of the percentage of muscularized pulmonary arteries in control or BLM-injected mice treated, or not, with ISO-1 or compound **31**. (**C**) Assessment of RV hypertrophy using the Fulton index (RV/(LV + S)) in control or BLM-injected mice treated, or not, with ISO-1 or compound **31**. Values are means ± SEM (*n* = 4–8), where *n* represents the number of mice. * p < 0.05; *** p < 0.001; **** p < 0.0001 compared to control mice. *# p =* 0.05; *## p <* 0.01 compared to BLM-injected mice. Scale bar = 50 μm in all sections. α-SMA = α-smooth muscle actin; AU = arbitrary unit; LV = left ventricle; RV = right ventricle; S = septum.

The murine model of BLM-induced pulmonary fibrosis is associated with the loss and remodeling of pulmonary arteries, two main characteristics of PH [15]. Since MIF is known to contribute to human and experimental PH [8,9,16,17], we next studied the effects of ISO-1 and compound **31** on the remodeling of the pulmonary vascular bed of BLM-injected mice (Figure 4). On day 21, substantial increases in the right ventricular systolic pressure (RVSP) and in the percentage of muscularized distal pulmonary arteries were found in BLM-injected mice treated with vehicle compared to control mice (Figure 4A,B). By contrast, significant reductions in the RVSP and the percentage of muscularized distal pulmonary arteries were noted in BLM-injected mice treated with ISO-1 and compound **31**, when compared to BLM-injected mice treated with vehicle (Figure 4A,B). Of note, no differences in the right ventricle (RV)/(left ventricle (LV) + septum (S)) ratio were found between the treated and untreated BLM-injected mice (Figure 4C). The presence of α-smooth muscle actin (α-SMA) positive cells around pulmonary vessels of BLM-injected mice treated with ISO-1 and compound **31** underline the fact that further studies are needed to identify the precise role of MIF and its actions, signaling the activation of fibroblast, transformation into myofibroblasts, as well as the production of extracellular matrix (ECM) components.

### 2.5. Chronic Treatment with ISO-1 and Compound ***31*** Decreases Perivascular Macrophage Accumulation in Lungs of Bleomycin-Injected Mice

**Figure 5:**
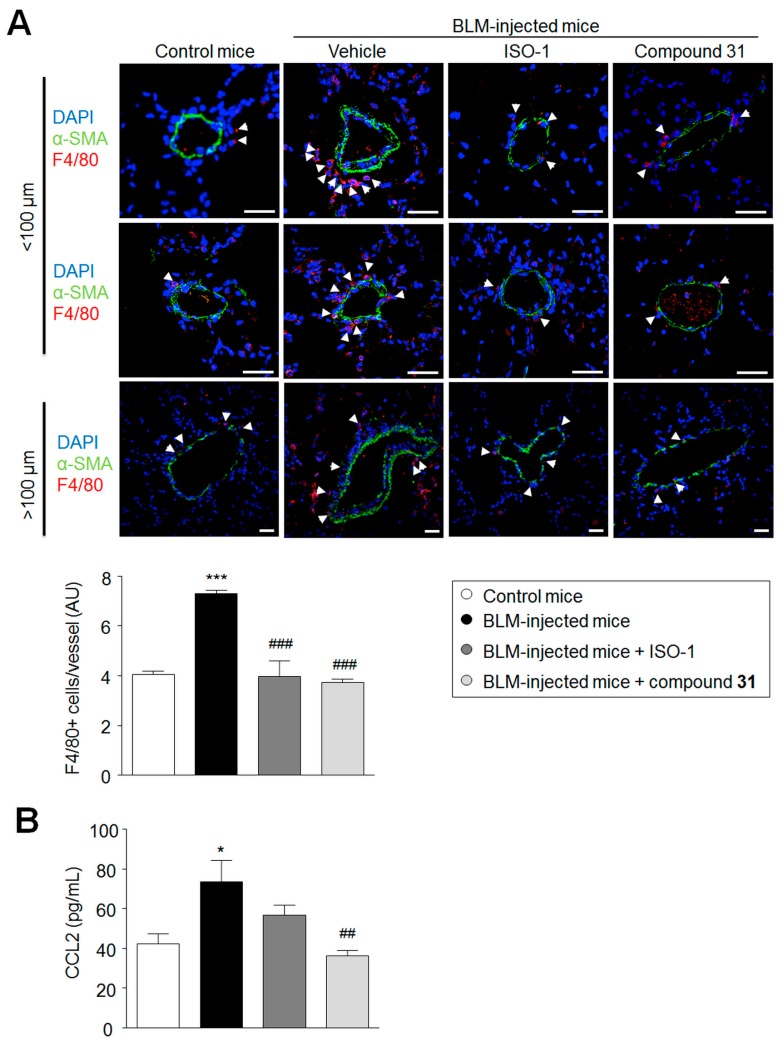
Chronic treatment with ISO-1 and compound **31** prevents the recruitment of perivascular macrophages in the lungs of bleomycin (BLM)-injected mice. (**A**) Representative images of F4/80 (white arrowhead) with α-smooth muscle actin (α-SMA; green) immunostaining and DAPI (blue) around vessels in lungs of BLM-injected mice treated, or not, with ISO-1 or compound **31**. (**B**) Levels of circulating CCL2 proteins in the serum of BLM-injected mice compared to those of control mice. Values are means ± SEM (*n* = 4–8), where *n* represents the number of mice. * p < 0.05; *** p < 0.001 compared to control mice. *## p <* 0.05; *### p <* 0.01 compared to BLM-injected mice. Scale bar = 20 μm in all sections. α-SMA = α-smooth muscle actin; AU = arbitrary unit.

MIF is a critical inflammatory mediator and, because inflammation plays an important role in bleomycin-injured lungs [18], we next examined the extent of macrophage infiltration in the lungs of BLM-injected mice treated with ISO-1 or compound **31** when compared to the lungs of BLM-injected mice treated with vehicle (Figure 5). The extent of F4/80+ macrophage infiltration was increased in the lungs of BLM-injected mice treated with vehicle when compared to control mice (Figure 5A). By contrast, a marked decrease in the extent of F4/80+ macrophage infiltration was observed in the lungs of BLM-injected mice treated with ISO-1 and compound **31** compared to the lungs of BLM-injected mice treated with vehicle (Figure 5A). Consistent with these findings, we observed that CCL2, a chemokine that is required for the recruitment of monocytes/macrophages [19], is substantially increased in the lungs of BLM-injected mice treated with vehicle when compared to control mice, but reduced in the lungs of BLM-injected mice treated with ISO-1 or compound **31** (Figure 5B).

## 3. Discussion

Unadapted remodeling of the pulmonary vascular bed is crucial for the development and progression of IPF [1,20]. This present translational study confirms a potential role for MIF in a murine model of BLM-induced pulmonary fibrosis. More intense immunoreactivity was noted for MIF, CD74, and CXCR4 in lungs from BLM-injected mice. Interestingly, these in vitro observations were also observed in lungs of IPF-PH patients, and confirmed by in vivo data showing that daily treatment with ISO-1 or compound **31**, starting 1 day after a single intratracheal injection of bleomycin, substantially attenuated the deposition of collagen and prevented pulmonary vascular remodeling and PH in mice. 

MIF is a pleiotropic protein that participates not only in the immune and inflammatory responses of many tissues and organs [21], but also in the synthesis and accumulation of the matrix [7,22,23,24] and in the vascular remodeling associated with PAH [8,9]. In accordance with this notion, the MIF rs755622*C allele was associated with susceptibility to PH in diffuse systemic sclerosis [25]. In the present study, our confocal microscopic analyses and double labeling with MIF, CXCR4, or CD74 indicated strong staining for these proteins in the paraffin-embedded lungs of patients with IPF-PH when compared to control subjects. Other clinical and translational studies have shown that MIF is also increased in the BAL and lungs of patients with IPF alone [4,5], suggesting that the upregulation of MIF precedes PH development. This notion is in line with our recent observations showing that CD74 and its signaling through MIF is at the crossroad of inflammation and pulmonary endothelial dysfunction in the pathogenesis of PAH [9]. Indeed, we have reported that pretreatment with exogenous MIF induces a substantial increase in secretions of endothelial-derived interleukin (IL)-6 and CCL2, as well as the expression of key adhesion molecules, leading to exaggerated peripheral blood mononuclear cell (PBMC) adhesion on confluent monolayers of endothelial cells derived from PAH patients and control subjects [9]. Therefore, further studies are needed to better understand the role of MIF and its signaling in IPF.

The targeting of the tautomerase enzymatic active site of MIF proved to be an attractive entry point for the design of small molecules that inhibit MIF receptor-mediated MIF biological activities [26,27]. For instance, the isoxazole derivative ISO-1 [28], that is reported to be among the first reversible MIF tautomerase inhibitors, has been shown to suppress the MIF biological activations of CD74 and CXCR4 [27,28]. However, although ISO-1 gained interest as a useful biological tool for the investigation of MIF-mediated signaling [29], it exhibited a moderate inhibition constant (Ki) value of 24 µM in the MIF tautomerase assay using 4-hydroxyphenylpyruvate (4-HPP) as substrate, and off-target effects have been demonstrated [30]. In the search of more potent, selective, and reversible MIF tautomerase inhibitors, several compounds displaying *K*i values in the submicromolar range were reported [31]. In this context, we recently identified that *N*-(3-hydroxy-4-fluorobenzyl)-5-trifluoromethylbenzoxazol-2-thione **31** (*K*i = 0.3–1µM) partially reversed established PH in a monocrotaline (MCT) rat model [6]. 

To evaluate the potential efficacy of ISO-1 and compound **31** against pulmonary vascular remodeling in the context of lung fibrosis, we used the well-characterized murine model of BLM [11,13,14]. A previous study from Tanino and colleagues [6] showed a substantial increase of MIF levels in the BAL and lungs of BLM-injected mice, starting 5 days after intratracheal administration of BLM. In this study, we also obtained evidence that MIF, CXCR4, and CD74 are overabundant, at day 21, in the lungs of BLM-injected mice when compared with the lungs of control mice. Tanino and colleagues [6] also reported that an anti-MIF antibody significantly attenuated BLM-induced acute lung inflammation, and significantly reduced mortality at day 14, as well as the histopathological lung injury score at day 10. However, treatment with the anti-MIF antibody was not found to attenuate the subsequent lung fibrosis in this model [6]. In contrast to the anti-MIF antibody, chronic treatments with the two small molecule MIF inhibitors, ISO-1 and compound **31**, reduced the accumulation of collagen in the lungs of BLM-injected mice. In addition, these two molecules prevent PH development in BLM-induced mice, with a trend toward better improvement with compound **31**. Although further studies are needed, the fact that MIF is stored in intracellular pools and has intracellular functions [32] may partially explain the difference in biological activity observed between anti-MIF antibodies and their small molecules counterparts.

Since MIF is a protein known to regulate macrophage accumulation at sites of inflammation, additional evaluations were performed to follow the extent of macrophage infiltration in the lungs of BLM-injected mice treated, or not, with ISO-1 and compound **31**. Consistent with the decrease in CCL2 levels resulting from chronic treatment with ISO-1 and compound **31**, a marked decrease in the extent of F4/80+ macrophage infiltration was observed in the lungs of BLM-injected mice treated with ISO-1 and compound **31**, compared to the lungs of BLM-injected mice treated with vehicle.

In summary, these findings underline that chronic treatments with small molecule MIF inhibitors ISO-1 or compound **31** attenuated BLM-induced lung inflammation and fibrosis, and prevented PH development in mice. These data provide evidence that the specific inhibition of MIF may represent a promising potential therapeutic avenue to prevent the development of PH in the setting of a fibrotic lung disease.

## 4. Materials and Methods

### 4.1. Study Population

This study was approved by the local ethics committee (CPP n 18.06.06 (19 July 2018) and n CO-08-003 (27 June 2008), CPP Est-III and CPP Ile-de-France VII, Le Kremlin-Bicêtre, France). All patients gave informed consent before the study. Diagnosis of IPF-PH was made according to the recently updated American Thoracic Society (ATS)/European Respiratory Society (ERS) consensus criteria [33]. All IPF-PH patients (*n* = 4) presented pulmonary lung fibrosis complicated by PH, diagnosed by right heart catheterization (mean pulmonary arterial pressure (mPAP) ≥25 mmHg at rest). Lung specimens obtained during lobectomy or pneumonectomy for localized lung cancer were used as controls. To rule out PH in these patients, preoperative echocardiography was performed. The lung specimens from the controls were collected at a distance from the tumor foci. Histopathological analysis retrospectively established the absence of tumor infiltration in all tissue sections.

### 4.2. Study Protocol

Mice (*n* = 32) were randomly divided into four groups. Mice were either treated by phosphate-buffered saline (PBS, control group) or bleomycin in monotherapy. Lung fibrosis was induced by a single intratracheal injection of bleomycin (3000 UI/kg) at day 0 (treatment group). Bleomycin-treated mice received either treatment with (*S*,*R*)-3-(4-hydroxyphenyl)-4,5-dihydro-5-isoxazole acetic acid methyl ester (ISO-1) or *N*-(3-hydroxy-4-fluorobenzyl)-5-trifluoromethylbenzoxazol-2-thione **31** [8] (20 mg/kg, once a day) per os from day 1 to day 21 after the administration of a single dose of bleomycin. The tested compounds were synthesized and kindly provided by the biotechnology company Apaxen, Gosselies, Belgium [8]. ISO-1 and compound **31** were dissolved in DMSO and stored, and then diluted with water (1:10) before their administrations by oral gavage.

### 4.3. Animals and Hemodynamic Measurements

Eight-week-old male C57BL/6 mice were purchased from Janvier Laboratory (Le Genest Saint Isle, France). Animals received humane care in compliance with the guidelines implemented at our institution (INSERM and University Paris Descartes. Ethics committee CEEA 34, protocol APAFIS2016033116014347).

Mice underwent hemodynamic evaluation at day 21 by right ventricular systolic pressure (RVSP) and heart rate measurement in unventilated mice under isoflurane anesthesia (1.5%–2.5%, 2 L O_2_/min) using a closed chest technique, by introducing a catheter (1.4 F catheter, Millar Instruments Inc, Houston, Texas) into the jugular vein and directing it to the right ventricle. After the completion of hemodynamic assessments, blood was collected by direct cardiac puncture and the mice were sacrificed by exsanguination. The heart and lungs were then removed en bloc, and right ventricular hypertrophy (RVH) was determined by the Fulton index measurement (right ventricle/left ventricle plus septum (RV/LV + S)). The pulmonary circulation was flushed with 5 mL of buffered saline at 37 °C, and then the left lung was prepared for morphometric analyses and the right lung was quickly harvested, immediately snap-frozen in liquid nitrogen, and kept at −80 °C for Western immunoblot analysis.

### 4.4. Immunostaining, Immunofluorescence, and Confocal Analyses

Immunohistochemistry and immunocytofluorescence staining for MIF, CD74, α-smooth muscle (SM) actin, or F4/80 were performed in human and mouse lung paraffin sections [34,35,36,37,38]. Briefly, lung sections (5 μm thickness) were deparaffined and stained with hematoxylin and eosin (Sigma-Aldrich, Saint-Quentin Fallavier, France), Sirius red, Masson’s trichrome, or incubated with retrieval buffer. Then, sections were saturated with blocking buffer (5% bovine serum albumin (BSA) (*w*/*v*) in phosphate-buffered saline (PBS)), and incubated overnight with specific antibodies, followed by corresponding secondary fluorescent-labeled antibodies (Thermo Fisher Scientific, Saint-Aubin, France). Nuclei were labeled using DAPI (Thermo Fisher Scientific). Mounting was done using ProLong Gold antifade reagent (Thermo Fisher Scientific). Images were taken using an LSM700 confocal microscope (Zeiss, Marly-le-Roi, France). Other lung sections were used for immunochemistry using a VECTASTAIN ABC kit, according to the manufacturer’s instructions (Abcys, Courtaboeuf, France), and counterstained with hematoxylin (Sigma-Aldrich). Images were taken using an Eclipse 80i microscope (Nikon Instruments, Champigny-sur-Marne, France). 

### 4.5. Second Harmonic Generation (SHG) Microscopy

Second harmonic generation microscopy offers the opportunity to image and quantify collagen without staining, and was used as previously described [39]. Briefly, a multiphoton inverted stand Leica SP5 microscope (Leica Microsystems GmbH, Wetzlar, Germany) was used for lung tissue imaging. A Ti:Sapphire Chameleon Ultra (Coherent, Saclay, France) with a center wavelength at 810 nm was used as the laser source for generating second harmonic (SHG) and two-photon-excited fluorescence (TPEF) signals. The laser beam was circularly polarized. A Leica Microsystems HCX IRAPO 25×/0.95 W objective was used to excite and collect SHG and TPEF signals.

Signals were detected in epi-collection through 405/15 nm and 525/50 bandpass filters, respectively, by NDD PMT detectors (Leica Microsystems) with a constant voltage supply, at constant laser excitation power, allowing the direct comparison of SHG intensity values. LAS software (Leica, Germany) was used for laser scanning control and image acquisition. Analyses were performed using a homemade ImageJ routine (http://imagej.nih.gov/ij/) as previously described [39]. Two fixed thresholds were chosen to distinguish biological material from the background signal (TPEF images) and specific collagen fibers (second harmonic generation (SHG) images). The SHG score was established by comparing the area occupied by the collagen relative to the sample surface. TPEF and SHG images were pseudocolored and overlaid for publication using ImageJ (V1.52a). 

### 4.6. Western Blot Analysis and ELISA

Lung tissues were homogenized and sonicated in RIPA buffer containing protease and phosphatase inhibitors, and 30 µg of protein was used for the detection of CXCR4, CD74, and GAPDH [36,37,38,40,41]. Concentrations of CCL2 in mice serum were evaluated using a specific ELISA kit (R&D Systems, Lille, France) according to the manufacturer’s instructions. 

### 4.7. Statistical Analyses

The data are expressed as means ± SEM. Statistical significance was tested using the nonparametric Mann–Whitney *U* test or two-way ANOVA with Bonferroni post hoc tests. Significant differences were assumed at a *p*-value < 0.05.

## Supplementary Materials

Supplementary materials can be found at http://www.mdpi.com/1422-0067/19/12/4105/s1.

## Abbreviations

AU	arbitrary unit
BLM	bleomycin
BAL	bronchoalveolar lavage
DAPI	4′,6-diamidino-2-phenylindole
H&E	hematoxylin and eosin
IPF	idiopathic pulmonary fibrosis
IL	interleukin
Ki	inhibition constant
PBMC	peripheral blood mononuclear cell
LV	left ventricle
α-SMA	α-smooth muscle actin
MIF	macrophage migration inhibitory factor
MCT	monocrotaline
PBS	phosphate-buffered saline
PH	pulmonary hypertension
PAH	pulmonary arterial hypertension
RV	right ventricle
RVH	right ventricular hypertrophy
S	septum
ISO-1	(*S*,*R*)-3-(4-hydroxyphenyl)-4,5-dihydro-5-isoxazole acetic acid methyl ester
4-HPP	4-hydroxyphenylpyruvate
**31**	*N*-(3-hydroxy-4-fluorobenzyl)-5-trifluoromethylbenzoxazol-2-thione **31**
