# Supplementary material for: Macrophage Migration Inhibitory Factor (MIF) Inhibition in a Murine Model of Bleomycin-Induced Pulmonary Fibrosis
Source: Int J Mol Sci. 2018 Dec 18;19(12):4105. doi: 10.3390/ijms19124105 (PMC6321607; doi:10.3390/ijms19124105)
Supplement: Supplementary file 1 [file ijms-19-04105-s001.pdf]

Supplementary: Macrophage Migration Inhibitory Factor (MIF) Inhibition in a Murine Model of Bleomycin-Induced Pulmonary Fibrosis

Sven Günther [1,2,3], Jennifer Bordenave [4,5], Thông Hua-Huy [1,2,3], Carole Nicco [1,2], Amélie Cumont [4,5], Raphaël Thuillet [4,5], Ly Tu [4,5], Timothée Quatremarre [4,5], Thomas Guilbert [1,2,6], Gaël Jalce [7], Frédéric Batteux [1,2], Marc Humbert [4,5,8], Laurent Savale [4,5,8], Christophe Guignabert [4,5,*,†] and Anh-Tuan Dinh-Xuan [1,2,3,*,†]

1 National Institute for Health and Medical Research (INSERM) UMR_S 1016, Cochin Institute, 75014 Paris, France; sven.gunther@aphp.fr (S.G); huythonghua@yahoo.com (T.H.-H.); carole.nicco@parisdescartes.fr (C.N.); thomas.guilbert@inserm.fr (T.G.); frederic.batteux@aphp.fr (F.B.); anh-tuan.dinh-xuan@aphp.fr (A.-T. D.X.)

2 Université Paris-Descartes, Sorbonne Paris Cité, 75014 Paris, France

3 Service de Physiologie-Explorations Fonctionnelles, Hôpital Cochin, Assistance Publique-Hôpitaux de Paris (AP-HP), 75014 Paris, France

4 INSERM UMR_S 999, Hôpital Marie Lannelongue, 92350 Le Plessis-Robinson, France; jennifer.bordenave@inserm.fr (J.B.); amelie.cumont@laposte.net (A.C.); raphael.thuillet@inserm.fr (R.T.); ly.tu@inserm.fr (L.T.); timothee.quatremare@inserm.fr (T.Q.); marc.humbert@bct.aphp.fr (M.H.); laurent.savale@gmail.com (L.S.)

5 Faculté de Médecine, Université Paris-Sud, Université Paris-Saclay, 94270 Le Kremlin-Bicêtre, France

6 National Centre for Scientific Research (CNRS) UMR 8104, 75014 Paris, France

7 Apaxen, 6041 Gosselies, Belgique; el.jalce@apaxen.com (G.J.)

8 Service de Pneumologie, Centre de Référence de l'Hypertension Pulmonaire, DHU Thorax Innovation, Hôpital Bicêtre, Assistance Publique-Hôpitaux de Paris (AP-HP), 94270 Le Kremlin-Bicêtre, France

* Correspondence: christophe.guignabert@inserm.fr; Tel: +33-1-40948833; Fax: +33-1-40942522;

† These authors contributed equally to this work.

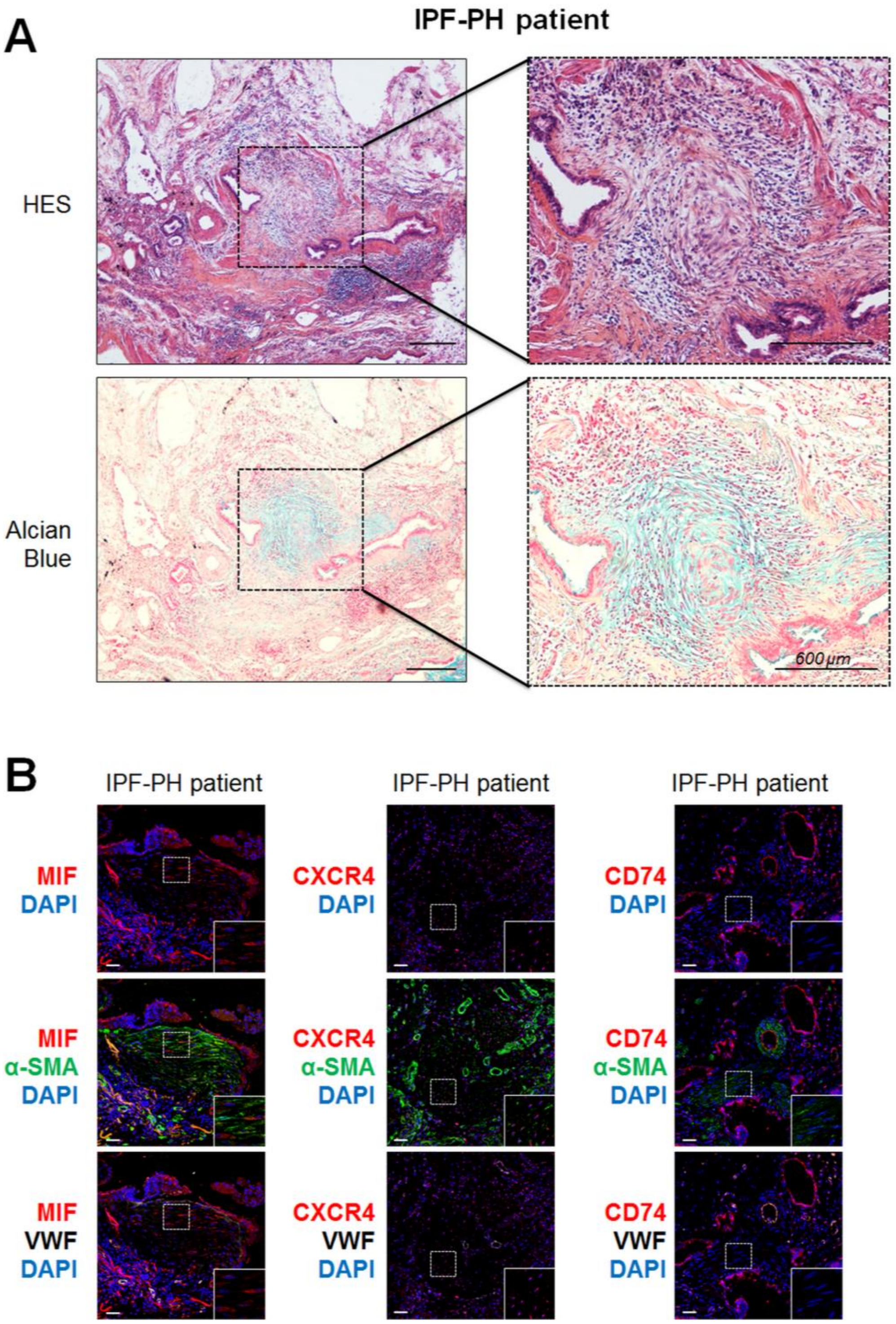

Figure S1. Expressions of MIF, CXCR4, and CD74 in fibrotic foci in lungs of patients with idiopathic pulmonary fibrosis with pulmonary hypertension (IPF-PH). **(A)** Alcian blue staining of a lung with IPF-PH fibroblastic foci. **(B)** Representative images of MIF (red; upper panel), CXCR4 (red; middle panel), and CD74 (red; lower panel) staining with α-smooth muscle actin (α-SMA; green) or von Willebrand factor (vWF; white) and DAPI (blue) in IPF-PH lungs (n = 3). Scale bar = 20 μm in all sections. DAPI = 4′,6-diamidino-2-phenylindole.
